# Periostin as a Biomarker of Allergic Inflammation in Atopic Bronchial Asthma and Allergic Rhinitis (a Pilot Study)

**DOI:** 10.17691/stm2020.12.5.04

**Published:** 2020-10-28

**Authors:** S.V. Krasilnikova, E.V. Tush, P.A. Frolov, D.Yu. Ovsyannikov, A.B. Terentyeva, N.I. Kubysheva, T.I. Eliseeva

**Affiliations:** Assistant, Department of Ear, Nose, and Throat; Privolzhsky Research Medical University, 10/1 Minin and Pozharsky Square, Nizhny Novgorod, 603005, Russia;; Associate Professor, Department of Hospital Pediatrics; Privolzhsky Research Medical University, 10/1 Minin and Pozharsky Square, Nizhny Novgorod, 603005, Russia;; Assistant, Department of Pediatrics; Peoples’ Friendship University of Russia (RUDN University), 6 Miklukho-Maklaya St., Moscow, 117198, Russia;; Head of the Department of Pediatrics; Peoples’ Friendship University of Russia (RUDN University), 6 Miklukho-Maklaya St., Moscow, 117198, Russia;; Associate Professor, Department of Ear, Nose, and Throat; Privolzhsky Research Medical University, 10/1 Minin and Pozharsky Square, Nizhny Novgorod, 603005, Russia;; Senior Researcher, Research Laboratory “Clinical Linguistics”; Kazan Federal University, 18 Kremlyovskaya St., Kazan, Republic of Tatarstan, 420008, Russia; Associate Professor, Professor, Department of Hospital Pediatrics Privolzhsky Research Medical University, 10/1 Minin and Pozharsky Square, Nizhny Novgorod, 603005, Russia;

**Keywords:** periostin, bronchial asthma, allergic rhinitis, chronic rhinosinusitis with polyps

## Abstract

The involvement of periostin in Th2-dependent allergic inflammation has been documented. However, the significance of periostin as a biomarker of local allergic inflammation in the nasal mucosa (NM) of patients with atopic bronchial asthma (BA) and allergic rhinitis (AR) is yet to be determined.

**The aim of the study** was to determine the presence of periostin and evaluate its role as a non-invasive marker of allergic inflammation in the nasal secretions of children with atopic BA and AR.

**Materials and Methods.:**

In 43 patients aged 4–17 years with atopic BA and AR, the NM was examined using nasal video-endoscopy and (if indicated) computed tomography; the amount of periostin in the nasal secretion was determined by the enzyme immunoassay.

**Results.:**

Exacerbation of AR was accompanied by a statistically significant increase in the level of periostin in the nasal secretion: up to 0.84 [0.06; 48.79] ng/mg, whereas in remission, that was 0.13 [0.00; 0.36] ng/mg; p=0.04. This value increased progressively as the severity of AR increased from 0.16 [0.00; 0.36] ng/mg in the mild course to 0.20 [0.00; 9.03] ng/mg in AR of moderate severity, and to 10.70 [0.56; 769.20] ng/mg in most severe cases; p=0.048. Hypertrophy or polyposis of the nasal and/or paranasal mucosa was detected in 34.9% (15/43) of the examined patients. The concentration of periostin in the nasal secretion was lower in children without NM hypertrophy: 0.18 [0.001; 4.30] ng/mg vs 0.78 [0.13; 162.10] ng/mg in patients with NM hypertrophy; the differences were close to statistically significant: p=0.051. The level of nasal periostin depended on the clinical form of hypertrophy in the sinonasal mucosa, reaching 0.17 [0.00; 0.32] ng/mg in children with polyposis hyperplasia of NM and 21.6 [10.70; 1516.80] ng/mg — with hypertrophy of the NM in the medial surface of the concha; p=0.02.

**Conclusion.:**

Exacerbation and increased severity of AR in patients with atopic BA are accompanied by an increased level of periostin in the nasal secretion. This allows us to consider the level of nasal periostin as a biomarker of local allergic inflammation in the NM of patients with atopic BA combined with AR. Hypertrophic changes in the sinonasal mucosa are generally accompanied by an increased level of nasal periostin; specifically, this level significantly depends on the clinical form of mucous membrane hypertrophy and requires additional studies.

## Introduction

Diseases of the upper respiratory tract (URT), including allergic rhinitis (AR) and allergic rhinosinusitis, are the most common comorbidities in patients with atopic bronchial asthma (BA) and can have a negative effect on its course. In this regard, current consensus documents recommend assessing the URT status at all stages of BA management [1–4].

The common pathogenetic mechanism of BA and allergic rhinitis/rhinosinusitis is allergic inflammation [1, 5, 6]. This may be associated with pathological remodeling of the respiratory tract, including the mucous membrane of the nose and paranasal sinuses [7]. Manifestations of URT remodeling in AR and allergic rhinosinusitis include epithelial hyperplasia, increased deposition and degradation of extracellular matrix components, and accumulation of plasma proteins [8]. The resulting hypertrophic changes in the sinonasal mucosa and the formation of polyps in the sinonasal zone aggravate the nasal obstruction, seriously impairing the quality of life of patients and having a negative impact on the achievement of asthma control [9–11].

Some authors point that hypertrophic and hyperplastic changes in the sinonasal mucosa are observed mainly in people over the age of 40 [12]. However, our studies and reports by others indicate that the debut of hypertrophy and hyperplasia can also occur in childhood, although there is no verified data on the prevalence of this pathology in children [3, 13, 14]. According to Karpova et al. [14], in children, polyposis-associated changes in the mucosa of the nose and paranasal sinuses are mainly due to AR.

In patients with allergic airway inflammation, no clear mechanism and risk factors for pathological remodeling of URT in hypertrophy and hyperplasia have been established until now [12]. The proposed hypotheses implicate the endotype and severity of allergic inflammation, which, in turn, are characterized by respective biomarkers [12, 15–17].

At present, at least two subtypes of inflammation have been described in BA and comorbid disorders of the URT: with a high activity of T-helper lymphocytes of the second type (Th2) and with a low activity of Th2 cells [12]. The subtype with a high Th2 response is the most common in BA patients, especially in children. It is associated with atopic syndrome and is characterized by a good response to therapy with topical glucocorticoids [12, 18, 19]. Th2-dependent allergic inflammation can trigger remodeling of the NM and contribute to hypertrophic and hyperplastic changes, including polyposis [20–23]. Patients with a Th2-dependent inflammatory endotype have the most severe and refractory course of the disease [24, 25].

Numerous cytokines are involved in inflammation of the Th2-dependent endotype. Among them are the so-called alarmins, initiators of Th2-inflammation: IL-33, IL-25, and thymic stromal lymphopoietin [26–29]. It is assumed that alarmins promote the expression of IL-4, IL-5, and IL-13, an increase in IgE antibody titers, eosinophilia, and increased levels of periostin. These cytokines are involved in the mechanism of the Th2-dependent inflammatory response, as well as in the associated pathological remodeling of the airways [12, 30]. It is, therefore, important to know biomarkers of allergic inflammation and pathological remodeling of the mucous membrane of the nose and paranasal sinuses in patients with BA, including children. Currently, periostin is recognized as a biomarker of advanced allergic inflammation of the second type [31].

Periostin is a matricellular protein with a molecular mass of 90 kDa; it is produced in response to inflammatory stimuli mediated by IL-4, IL-5, and IL-3 by many cells, including epithelial cells and fibroblasts [7, 32]. There is evidence that periostin modulates URT inflammation and remodeling, but its precise functions are not fully understood [33]. Periostin is able to induce the differentiation of fibroblasts into myofibroblasts and enhance fibrosis by binding to other proteins of the extracellular matrix, such as type I collagen, fibronectin, and tenascin C, as well as by inducing collagen fibrillogenesis and cross-linking [34]. Periostin can influence epithelial remodeling, promoting epithelial-mesenchymal transition [34], in which cells of the respiratory epithelium gradually transform into mesenchymal cells in the process of developing fibrosis [35]. Basal secretion of periostin by epithelial cells can change the underlying matrix by modifying the deposition and crosslinking of collagen fibrils [34].

Thus, considering the available data, periostin can be called a recognized biomarker of allergic inflammation of the second type and a potential marker of the associated pathological remodeling. It should be noted that most of the data on the role of periostin in inflammation of the second type and pathological remodeling in patients with chronic allergic airway diseases were obtained by invasive techniques, i.e., by studying tissue biopsies and/or determination of periostin in blood serum [33–35].

Until now, the non-invasive quantification of periostin in bio-substrates (for example, in nasal secretions) remained insufficiently developed. There are only isolated reports on the measurement of periostin in nasal secretions of patients with atopic asthma and nasal symptoms. We found a study by Pham et al. [36], which demonstrated abnormally high levels of nasal periostin in patients with asthma that increased even more with asthma exacerbation. In those cases when BA and AR were combined, the levels of nasal periostin were significantly higher than in patients with asthma alone (520.19 and 4.71 pg/ml, respectively; p<0.05). However, this work did not analyze relations between the severity of nasal symptoms and the amount of nasal periostin and did not consider the role of hypertrophic changes in the sinonasal mucosa.

Studying the presence of periostin in the nasal secretion of patients with atopic asthma and nasal symptoms is essential to determine the value of periostin as a non-invasive marker of type 2 allergic inflammation and the associated pathological remodeling of the airways.

**The aim of the study** was to measure the level of periostin and evaluate its role as a marker of allergic inflammation in the nasal secretions of children with atopic bronchial asthma combined with allergic rhinitis, considering the clinical manifestations and hypertrophic changes in the mucosa of the nose and/or paranasal sinuses.

## Materials and Methods

This pilot non-randomized single-center observational study was conducted in accordance with the requirements of the Helsinki Declaration (2013). The protocol was approved by the Ethics Committee of the Privolzhsky Research Medical University. Informed consent was obtained from patients aged 15 to 17 years and from the parents of children under 15 years old in accordance with Federal Law No.323 of November 21, 2011 “On the basics of protecting the health of citizens in the Russian Federation”.

The study included 43 children aged 4 to 17 years (average age — 9.0 [7.0; 13.0] years); among them, boys — 67% (29/43), girls — 33% (14/43). The children underwent treatment for atopic BA and AR in the Children’s City Clinical Hospital No.1 of Nizhny Novgorod in 2019–2020. The study inclusion criterion was the diagnosis of asthma made in accordance with the current international and national recommendations [1]. The exclusion criteria were acute infectious diseases, fever, diabetes mellitus, autoimmune disorders, primary immuno-deficiencies, oncological diseases, and oral administration of glucocorticoids [37]. The diagnosis and severity of asthma were determined by the attending physician according to the GINA recommendations available at that time [1]. Treatment of BA and AR was carried out by the accepted methodology [1, 38].

All children underwent general clinical examination and assessment of susceptibility to antigens by detecting IgE antibodies or by skin tests to common allergens known to cause allergies in populations of the Volga-Vyatka region of Russia [39]. Routine examination of the ENT organs, rhino video endoscopy using flexible optics with 3.2 mm outer diameter (Atmos, Germany), as well as CT of the paranasal sinuses (when indicated) were performed [5]. The Total Nasal Symptom Score (TNSS) scale was used to assess the severity of the nasal symptoms [40]. The Asthma Control Questionnaire-5 (ACQ-5) was used to quantify the level of BA control [41].

Collection of nasal secretion specimens for subsequent determination of periostin was carried out using Merocel spongy swabs (Medtronic, USA). Pre-weighed swabs were placed under the mid-concha on each side of the nose for 2 min. Then, the swabs with the absorbed nasal secretion were re-weighed and mixed with 1.5 ml of saline in a tube [42]. The tube was placed in a shaker for 10 min, then centrifuged at 1500 rpm for 10 min. The collected supernatant was stored at –20°C for no longer than two weeks. Periostin in the nasal secretion was quantified using the Human Periostin/ OSF-2 ELISA Kit (Aviscera Bioscience, Inc., USA) and an ALISEI Quality system automatic immunoassay analyzer (Radim, Italy) [43]. In accordance with the manufacturer’s instructions, the measurement was performed twice for each patient (paired wells). For the subsequent analysis, the mean of the two values was used. The periostin content was expressed in nanograms per 1 mg of nasal secretion.

**Statistical processing of the data.** The study was pilot in nature; therefore, no minimal sample size required for statistical significance was calculated. The statistical analysis was performed using Statgraphics Centurion package, v. 16.1.17. To determine the normality of data distribution for quantitative comparison, standardized skewness and standardized kurtosis were calculated. If the calculated values of these indicators were outside the range –2 ... +2, then the original data sets were considered different from normal. For the principal analyzed parameter — concentration of periostin in the nasal secretion — the standardized asymmetry and standardized kurtosis exceeded a value of +5. Thus, we considered the periostin data distribution as different from the normal one; accordingly, we applied nonparametric tests to determine the differences between the groups. For two compared groups, this was the Wilcoxon W-test (Mann–Whitney) — a comparison of the medians of the two data samples; here, the data are presented as Me [Q1; Q3], where Мe is the median value and [Q1; Q3] are the first and third quartiles. The Kruskal–Wallis test (KW test) was used to compare the medians between several groups. The relationship between the parameters was assessed using Spearman’s rank correlation (p). Differences between qualitative characteristics were analyzed using the c^2^ criterion. The differences were considered statistically significant at p<0.05.

## Results

### Clinical characteristics of the patients.

 The median age of the examined children was 9.0 [7.0; 13.0] years, boys and girls were comparable in age. All the examined patients with atopic BA showed signs of AR. In 14% (6/43) of children, the AR course was mild, in 67.4% (29/43) — moderate, and in 18.6% (8/43) — severe. In the majority (81.4%, 35/43) of patients, AR was characterized by a persistent course, whereas an intermittent course was noted in 18.6% (8/43) of children. AR in remission was diagnosed in 30.2% (13/43) of patients and exacerbated AR — in 69.8% (30/43).

Most of the patients had comorbid URT disorders. Thus, according to the results of video endoscopic examination, 34.9% (15/43) of children showed hypertrophy of the nasal mucous membrane of varying severity: from local hypertrophy of the mucous membrane of the medial surface of the concha (n=9) to polyposis extending to the paranasal sinuses (n=6). Children with hypertrophic changes were slightly older than those without them (W=281.0; p=0.07), no significant gender differences were found (W=211.5; p=0.6). The course of AR in children with NM hypertrophic changes was moderate to severe in 46.7% (7/15) of cases, severe in 53.3% (8/15). Among patients with mild AR, no hypertrophic changes in the sinonasal mucosa were detected. Thus, hypertrophic changes in the NM were significantly more frequent in patients with moderate and severe AR (c^2^=19.6; p=0.001). Hypertrophy of the pharyngeal tonsil was noted in 72% (31/43) of children; anomalies of intranasal structures, mainly, the curvature of the nasal septum, were found in 72% (31/43) of children.

The severity of nasal symptoms in children with BA and AR according to the TNSS averaged at 5.0 [3.0; 8.0] points. These values were changing in the course of AR: from 3.0 [2.0; 5.0] points in remission to 6.0 [4.0; 8.0] points in the period of AR exacerbation (W=316.0; р=0.001) (Table 1). The rhinoscopic picture of AR in children with BA was characterized by a changed color of the NM, the presence or absence of edema, and discharge in the nasal passages. There was a trend to lower TNSS values in patients with physiological color of NM: 2.5 [0.0; 5.0] points; in patients with pale NM in the nasal concha, the score was 5.0 [3.0; 7.0], and in patients with hyperemia — 7.0 [6.0; 9.5] (KW=5.1; p=0.08).

**Table 1:** Periostin content in nasal secretions of children with atopic bronchial asthma and allergic rhinitis (Me [Q1; Q3])

Indicators	All patients (n=43)	Period of allergic rhinitis		Statistical differences between groups
		Remission (n=13)	Exacerbation (n=30)	
TNSS (points)	5.0 [3.0; 7.0]	3.0 [2.0; 5.0]	6.0 [4.5; 8.5]	W=360.5; p=0.0001
Periostin (ng/mg)	0.28 [0.01; 10.69]	0.13 [0.00; 0.36]	0.84 [0.06; 48.79]	W=274.0; p=0.04

### Periostin in nasal secretions of patients with different clinical courses of allergic rhinitis.

 The median concentration of periostin in nasal secretions of children with atopic BA and AR was 0.28 [0.01; 10.69] ng/mg (see Table 1). No correlation of this parameter with age (R=0.04; p=0.78) or gender (W=174.0; p=0.46) of patients was found.

The concentration of periostin in the nasal secretion correlated with the period of AR: it was significantly higher in patients with AR exacerbation: 0.84 [0.06; 48.79] ng/mg, whereas in remission, it was 0.13 [0.00; 0.36] ng/mg (W=274.0; p=0.04). In addition, the levels of periostin significantly correlated with the severity of AR: the levels as low as 0.16 [0.00; 0.36] ng/mg were found in children with mild AR (n=6) and the maximal values 10.70 [0.56; 769.20] ng/mg were found in children with a severe course of the disease (n=8). In AR of moderate severity (n=29), the concentration of periostin was 0.20 [0.00; 9.03] ng/mg (KW=6.1; p=0.048).

An additional correlation was found between the level of periostin in the nasal secretion and the total severity of nasal symptoms, assessed by the TNSS scale: R=0.31 at p=0.04 (Table 2). Thus, an aggravation of the AR symptoms was accompanied by a progressive increase in the content of periostin in the nasal secretion.

**Table 2:** Correlations between the severity of symptoms of allergic rhinitis (TNSS scale, points) and the level of periostin in nasal secretions

Clinical parameter	R	p
Rhinorrhea	**0.36**	**0.02**
Itching	0.16	0.32
Sneezing	0.13	0.39
Obstruction	0.21	0.19
TNSS	**0.31**	**0.04**

We also analyzed the relationship between the presence of periostin in the nasal secretion and the severity of individual AR symptoms (see Table 2). A direct correlation was detected between the level of periostin in the nasal secretion and the severity of rhinorrhea: R=0.36; p=0.02. The severity of itching, sneezing, or nasal obstruction had no significant correlation with the nasal periostin under the given conditions.

### Periostin in nasal secretions of patients with hypertrophy of the sinonasal mucosa.

 Our study showed that patients with hypertrophic changes in the sinonasal mucosa (n=15) had higher levels of periostin in the nasal secretion: 0.78 [0.13; 162.10] ng/mg compared with children without NM hypertrophy (n=28): 0.18 [0.001; 4.30] ng/mg; the differences were close to significant: W=286.5; p=0.051.

**Figure 1:**
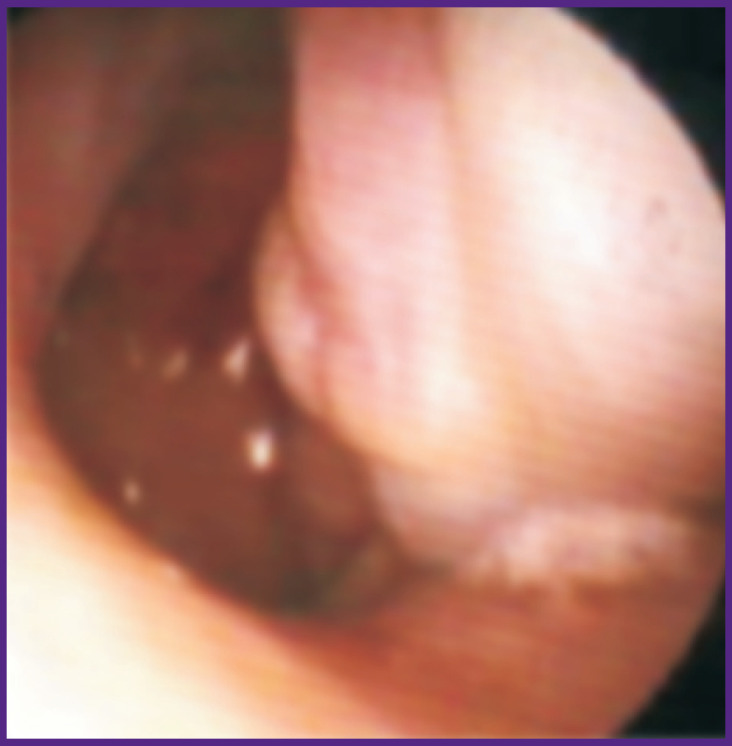
Fragment of nasal video-endoscopy of patient S., 8 years old, diagnosed with bronchial asthma (atopic, persistent, under control), allergic rhinitis (persistent, severe) in the stage of exacerbation, and hypertrophy of the mucous membrane in the posterior parts of the inferior conchae Hypertrophic changes in the mucous membrane of the posterior ends of the inferior conchae. Periostin level in the nasal secretion is 10.7 ng/mg

**Figure 2:**
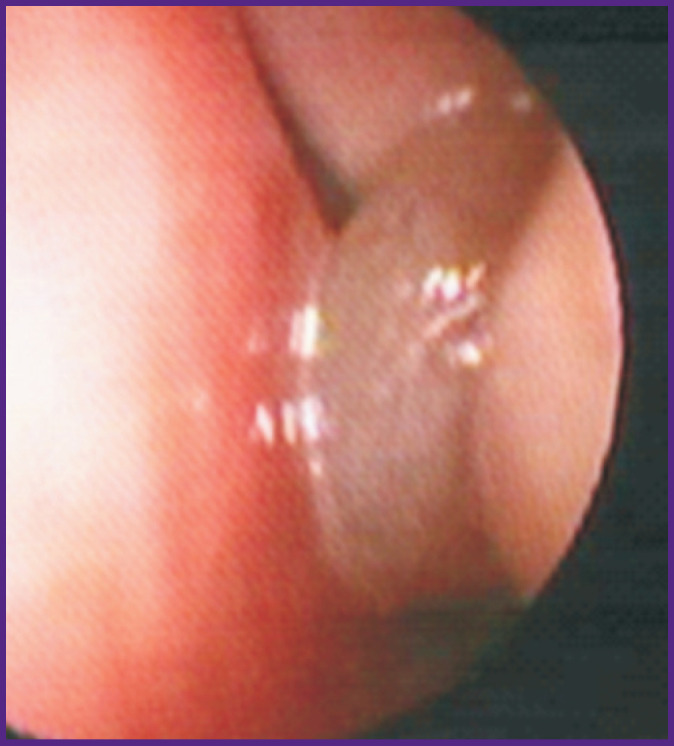
Fragment of nasal video-endoscopy of patient M., 9 years old, diagnosed with bronchial asthma (atopic, mild, intermittent) in remission, allergic rhinosinusitis (persistent), and polypoid rhinosinusitis Polyposis associated hypertrophy of the mucous membrane that obturates the middle nasal passage on the left. Periostin level in the nasal secretion is 0.056 ng/mg

We then analyzed changes in the periostin levels in various clinical forms of NM hypertrophy. In 9 patients with BA and AR, there was hypertrophy of the mucous membrane in the medial surface of the concha; in 6 patients, there was polyposis of the NM, extending to the paranasal sinuses. The level of periostin in the nasal secretion of children with hypertrophies (n=9) was 21.6 [10.7; 1516.8] ng/mg, which was significantly higher than that in children with polyposis (n=6): 0.17 [0.001; 0.32] ng/mg; W=48.0; p=0.02 (Figures 1, 2).

In figures, fragments of nasal video-endoscopy in patients with BA and AR who had various hyperplastic and hypertrophic changes in the sinonasal mucosa are shown. Notably, in a female patient with atopic BA and AR who had NM hypertrophy in the medial surface of the posterior ends of the inferior conchae, the level of periostin in the nasal secretion was high: 10.7 ng/mg (see Figure 1). Along with that, in another female patient with atopic BA and AR who had polyposis-associated hypertrophy of the NM (with obstruction of the middle nasal passage on the left), the level of nasal periostin was significantly lower: 0.056 ng/mg (see Figure 2).

We found no significant correlation between the level of periostin and changes in the nasal architectonics or the state of the pharyngeal tonsil. Thus, the median values of periostin in patients with normal nasal architectonics (n=12) were 0.15 [0.001; 0.63] ng/mg, and in children with anomalies of intranasal structures (n=21) — 0.45 [0.001; 21.59] ng/mg (W=242.5; p=0.13). In children with a normally sized pharyngeal tonsil (n=13), the average periostin value was 0.27 [0.16; 0.81] ng/mg, whereas in patients with a hypertrophic tonsil (n=20) that was 0.28 [0.001; 13.28] ng/mg (W=173.0; p=0.74).

## Discussion

The study was the first to compare the levels of periostin in the nasal secretion of children with atopic bronchial asthma and various clinical forms of allergic rhinitis. We also aimed to assess the potential significance of nasal periostin as a biomarker of allergic inflammation of the URT and hypertrophic changes in the sinonasal mucosa.

We found that the clinical period of AR determined, to a large degree, the presence of periostin in the nasal secretion of patients. Thus, in the periods of AR exacerbation, the levels of periostin were significantly higher than those during the periods of remission. This is consistent with reports by others indicating that the developing allergic inflammation in the airways is accompanied by an increase in the synthesis of periostin. Lopez-Guisa et al. [44] demonstrated that the expression of periostin genes by nasal epithelial cells after stimulation with IL-4/IL-13 was significantly (3.9-fold) higher in BA patients as compared with non-asthmatics. In a study by Pham et al. [36], abnormally high levels of nasal periostin were found in patients with asthma, and a further increase in periostin was noted during asthma exacerbation.

Likewise, in the present study, an aggravation of AR in patients with asthma was accompanied by a significant accumulation of periostin in nasal secretions; p=0.048. Quantification of AR symptoms using the TNSS scale revealed a significant positive correlation between the TNSS scores and the concentrations of periostin in nasal secretions (R=0.31; p=0.04). Among the symptoms, rhinorrhea best correlated with periostin levels (R=0.36; p=0.02). This result is consistent with the available literature in that an increased expression of periostin correlates with an increased activity of the glands of the respiratory tract and an increased volume of their secretion [33, 45].

Thus, if we consider the clinical manifestations of AR as a reflection of allergic inflammation, it can be argued that an intensification of the inflammatory process is accompanied by an increased accumulation of periostin in the nasal secretion. Therefore, the level of nasal periostin can be proposed as a non-invasive biomarker of allergic inflammation of the nasal mucosa in patients with atopic BA combined with AR. This idea is supported by the available data on the involvement of periostin in the genesis of allergic Th2-dependent inflammation in the respiratory tract [31, 33, 46, 47].

Regarding the relation between periostin and hypertrophy of the sinonasal mucosa, our results were less definitive. On the one hand, the level of nasal periostin showed some increase in patients with hypertrophic changes in the NM. On the other hand, this level significantly varied with the clinical variant of sinonasal hypertrophy. In patients with polyposis of the sinonasal mucosa, it was 0.17 [0.00; 0.32] ng/mg, i.e., close to the level of nasal periostin in patients without hypertrophy (0.18 [0.00; 4.30] ng/mg; p=0.83). Furthermore, in children with NM hypertrophy in the medial surface of the conchae, there was significantly more periostin, i.e., 21.6 [10.7; 1516.8] ng/mg; p=0.02. This, however, contradicts the literature data about an increased expression of the gene encoding for periostin and an increase in periostin-protein in sinonasal polyps and systemic bio-substrates [30, 45, 46]. For example, in a study by Asano et al. [48], serum periostin levels in patients with asthma with chronic rhinosinusitis and nasal polyps were significantly higher (130.0±46.6 ng/ml) than in patients without nasal polyps (87.9±37.7 ng/ml); p=0.001. However, they determined periostin in the blood serum, which is a systemic bio-substrate. Lehmann et al. [49] also provide numerous data on increased levels of periostin-protein and the expression of the respective gene in nasal polyps in patients with Th2-dependent inflammation and/or atopic BA.

In our view, this contradiction can be explained by that that the periostin-specific remodeling effects manifest to a higher degree when periostin secretion is directed towards the basement membrane. Additionally, in some cases of pathological remodeling, an increased presence of periostin is more typical of body tissues and systemic bio-substrates, while the accumulation of periostin in local secretions is less pronounced [34].

The major results of this study indicate that an increased level of periostin in the nasal secretion in patients with BA and AR is characteristic of an exacerbation of AR and an increase in the severity of its course. This allows us to consider the concentration of periostin in the nasal secretion as a biomarker of local allergic inflammation of the URT in these patients.

At the same time, the informativity of this biomarker may be ambiguous as far as the hypertrophic changes in the sinonasal mucosa are concerned. The levels of periostin in the nasal secretion of children without sinonasal hypertrophy and children with sinonasal polyposis were close, but significantly lower than those in children with hyperplasia of the mucous membrane of the medial surfaces of the conchae. This fact does not allow us to consider the nasal level of periostin a universal biomarker of sinonasal hypertrophy and requires additional research. It is quite possible that the use of minimally invasive techniques, including a brush biopsy, will make it possible to better assess the informativity of determining periostin as a biomarker of hypertrophic changes in the sinonasal mucosa.

Limitations of this study included the insufficient sample sizes (the numbers of patients) in the compared groups that did not provide for full-fledged statistical analysis. Yet, this work is the first to quantify the level of periostin in the nasal secretion of children with atopic BA and AR and to relate those to the clinical characteristics of the patients. The obtained results lay the ground for further studies using the required sample size and precisely quantify the levels of periostin in the nasal secretion in these patients. At the next stage of the research, it is planned to study the relationship between the level of periostin in the nasal secretion and other biomarkers of inflammation and tissue remodeling in patients with BA and AR.

## Conclusion

Exacerbation and increased severity of AR in patients with atopic BA are accompanied by an increase in the concentration of periostin in the nasal secretion. This allows us to consider the level of nasal periostin as a non-invasive biomarker of local allergic inflammation of the nasal mucosa in patients with atopic BA and AR. The accumulation of periostin in a hypertrophic sinonasal mucosa may reflect the clinical form of the hypertrophy and requires additional study.
